# Towards a Scalable Production of *Staphylococcus epidermidis* Extracellular Vesicles: Integration of Stirred-Tank Bioreactor and Ultrafiltration-Based Recovery

**DOI:** 10.3390/ijms27177797

**Published:** 2026-08-31

**Authors:** Giacomo Presutti, Francesca Bosco, Alessandro Chiadò, Tania Limongi, Marta Vallino, Roberto Pisano

**Affiliations:** 1 Department of Applied Science and Technology, Politecnico di Torino, 24 Corso Duca Degli Abruzzi, 10129 Torino, Italy; giacomo.presutti@polito.it (G.P.); francesca.bosco@polito.it (F.B.);; 2 Department of Drug Science and Technology, University of Torino, Via Pietro Giuria 9, 10125 Torino, Italy; 3 Consiglio Nazionale delle Ricerche (CNR) di Torino, Strada delle Cacce 73, 10129 Torino, Italy

**Keywords:** Gram-positive, extracellular vesicles, scale-up, ultrafiltration, ultracentrifugation

## Abstract

Extracellular vesicles (EVs) from Gram-positive bacteria are gaining more interest as potential biotechnological systems for vaccines and drug delivery. However, scalable production protocols remain poorly defined. Here, we evaluated upstream and downstream processes for the controlled production of extracellular vesicles from *Staphylococcus epidermidis*. Bacterial growth and vesicle release were compared between shake-flask cultures and a controlled stirred-tank bioreactor (STBR), and vesicles were isolated using either ultracentrifugation (UC) or ultrafiltration (UF). STBR cultivation significantly increased biomass accumulation during the exponential phase while preserving the same growth observed in shake flasks. Nanoparticle tracking analysis (NTA) showed time-dependent particle accumulation in both systems, with UF consistently yielding higher particle recovery than UC. Despite differences in yield, vesicles isolated by both methods displayed comparable size distributions and TEM imaging confirmed the spherical morphology. Protein quantification revealed greater protein recovery in UF preparations, whereas particle-to-protein ratios indicated similar sample composition between isolation methods. Together, these results suggest that controlled STBR fermentation combined with UF enables a reproducible, scalable production of *S. epidermidis* EVs without compromising vesicle integrity. This work provides an integrated framework for Gram-positive EV manufacturing and supports future translational applications.

## 1. Introduction

Bacterial extracellular vesicles (EVs) are nano-sized membrane-derived particles composed of a phospholipid bilayer. They have a diameter ranging from 40 to 400 nm, depending on the bacterial strain, and contain a plethora of bacteria-derived components such as nucleic acids, cytosolic proteins, antigens, and endotoxins [1]. They play an essential role in several bacterial processes like nutrient absorption, antibiotic resistance, cell–cell interaction, biofilm formation and delivery of virulence factors [2,3,4]. EVs can be released from both Gram-negative (often called Outer Membrane Vesicles, OMVs) and Gram-positive (often called Cytoplasmic Membrane Vesicles, CMVs) bacteria with different mechanisms of production, and they differ in their architecture, structure and components [1,5,6]. EVs from Gram-positive bacteria, and in particular from the *Staphylococcus* genus, have been explored for less than two decades [7], and studies in this field are still limited [8,9,10]. This is due to the structural differences between Gram-positive and Gram-negative bacteria cell wall. The thick peptidoglycan outer layer of the former was thought to interfere with the biogenesis of CMVs, thus preventing a thorough isolation. CMV biogenesis has been associated with different mechanisms including localized endolysin-mediated peptidoglycan degradation and, in some cases, explosive cell lysis [11]; however, accumulating evidence suggests that vesicle release can also occur in viable cells through regulated processes [12,13,14]. Bacterial vesiculation is influenced by a plethora of factors including bacteria strain and physiology, culture conditions and downstream procedures. As per the growth conditions, different media [15], temperature [16], pH [12], or antibiotic stress [17,18,19] can all modulate EV yield, morphology and cargo. Up to now, the most common way to cultivate bacteria for EVs production in liquid media has been the shake-flask culture, which is easy to use and highly versatile. However, it provides limited control over key cultivation parameters, including pH and dissolved oxygen levels, potentially affecting culture reproducibility and hindering process standardization [20]. Furthermore, shake flasks are characterized by a limited Oxygen Transfer Rate (OTR) since the mass transfer mainly occurs through the liquid surface and along the flask walls [21]. Scalable production of bacterial EVs remains a major bottleneck in the field, thus the use of bioreactors may be considered. Indeed, they enable the processing of a much larger volume of media, precisely controlling culture conditions such as pH and pO_2_ levels, along with temperature and mixing that can also be controlled in flask culture, although to a lesser extent. Moreover, since bioreactors employ an active aeration of the culture through a sparger, they have a higher OTR and overall better growth [22,23,24]. The other critical factors that can influence EV production and yield are the isolation and purification methods. Due to the high diversity in bacterial morphology, structure, metabolism, and biology, there is no standardized protocol to process culture media and extract EVs [25], although some common steps include an initial centrifugation to separate the biomass, a series of filtrations with different cutoffs to eliminate all the cellular debris and a final ultracentrifugation (UC) to obtain the EV sample [25,26]. The lack of standardized workflows contributes to variability in EV yield, composition, and functional properties across studies. UC is the most widely used technique for EV isolation, but it has some major drawbacks that limit the scale up of the process. Firstly, it is not easily scalable to large sample volumes, even at the pilot scale. Indeed, it processes only small volumes at a time and is a highly time- and energy-intensive method that requires complex manual procedures [27,28,29,30]. Furthermore, the high shear forces may lead to vesicles’ aggregation, damage or disruption, thus decreasing the purity and the yield of the sample [31]. Ultrafiltration (UF) could be used as an alternative to obtain EV-enriched samples, due to its ability to separate particles and other components based on their size or molecular weight. Centrifugal force, pressure or vacuum, is applied to filter the EV-rich supernatant through membranes of different cutoffs (ranging from 10 to 300 kDa) and does not require expensive and time-consuming equipment [26,27,32]. Furthermore, compared with ultracentrifugation, UF is markedly faster, simpler to implement, and associated with lower costs and reduced energy consumption [33]. Although shear stress is present during UF, its magnitude strongly depends on membrane geometry and operating conditions. Membrane-surface shear stresses of 1.33–1.48 Pa have been reported for a vibratory hollow-fiber system [34], whereas an EV-specific tangential-flow filtration study showed that shear stresses above 9.05 Pa caused protein aggregation and damage to small extracellular vesicles [35]. These process-level membrane shear stresses are not directly equivalent to the particle-surface stresses experienced during UC (Table S3). Nevertheless, as shown in the Supporting Information (Table S1), modeling a 150 nm vesicle as a rigid sphere with a density of 1.045 g mL^−1^ sedimenting in water at 25 °C under 100,000× *g* yields a maximum tangential surface shear stress of 1.18 Pa and a surface-area-averaged magnitude of 0.92 Pa in the dilute Stokes-flow limit [36,37,38,39]. In a freely sedimenting concentrated suspension, the Richardson–Zaki relation predicts hindered sedimentation as follows: the particle velocity decreases with increasing particles (Table S2). An ultrafiltration strategy could then be suitable for a scale up of the process, allowing for a higher yield of the final product. In this context, the UF can be exploited as Tangential Flow Filtration (TFF), in which a stream of fluid containing EVs flows tangentially across a membrane with a specific molecular weight cutoff (MWCO). Molecules smaller than the MWCO value pass through the membrane and go in the permeate, while bigger particles remain in the retentate, get recirculated and concentrated, thus reducing the membrane fouling compared to a classical dead-end system [27,40]. Several studies focused on the scale up of the OMV isolation process [22,41,42,43], but to date integrated upstream–downstream optimization for Gram-positive EV production has not been thoroughly addressed. *S. epidermidis*-derived EVs have recently attracted interest for their potential biomedical applications, particularly due to the immunomodulatory activity demonstrated by vesicles from commensal strains in preclinical models of inflammatory skin diseases, including psoriasis and atopic dermatitis [44,45]. Moreover, they have shown antibacterial and anti-biofilm activity against methicillin-resistant *S. aureus* (MRSA), suggesting their potential use in the treatment of severe bacterial infections [46]. Their potential as natural nanocarriers has also recently been demonstrated by Zhou et al. [47], who successfully encapsulated levofloxacin, resulting in an EV-based nanoantibiotic platform for the treatment of *S. aureus*-associated atopic dermatitis. These findings, together with the broader development of bacterial EVs as drug-delivery and therapeutic platforms [5], highlight the increasing interest in *S. epidermidis* EVs beyond basic biological studies. However, translation toward biomedical applications requires reproducible production and recovery of larger EV quantities, emphasizing the need to develop controlled cultivation and downstream processing strategies that can ultimately be transferred to larger scales. In this study, we optimized the culture conditions of *S. epidermidis* for EV isolation in a stirred-tank bioreactor (STBR) to process large volumes as a first scale-up strategy for a high-yield particle production. Furthermore, a side-by-side comparison between UC and dead-end UF is performed and their influence on particle size and morphology is assessed. These findings will foster the translation of bacterial EVs into clinical and industrial applications, including vaccine development and drug delivery.

## 2. Results

### 2.1. Growth Curves and Biomass Evaluation

**Figure 1:**
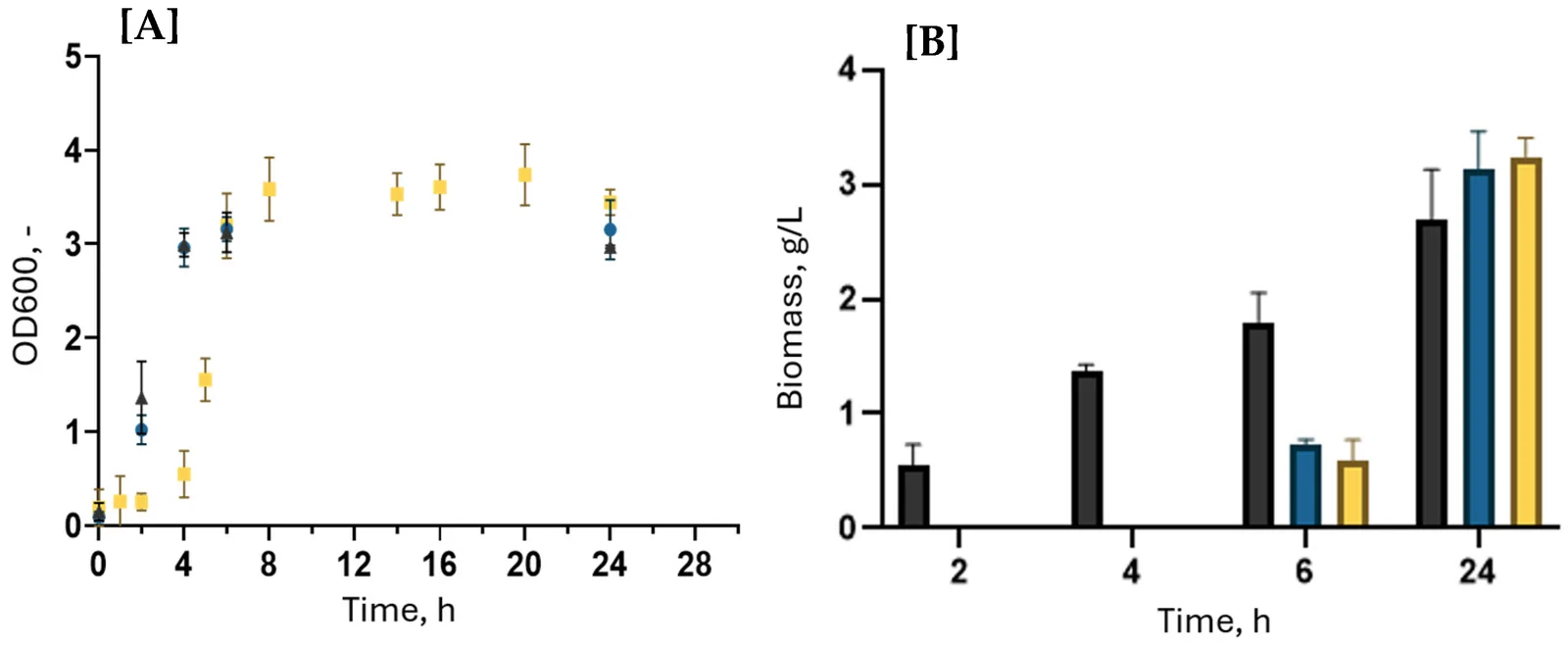
Comparison of (**A**) growth curves and (**B**) biomass dry weight between the STBR and the flask culture of *S. epidermidis*. 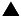 = STBR; 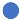 = flask with preculture; 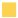 = flask without preculture.

To optimize the upstream process, the effect of a preculture step on the bacterial growth of *S. epidermidis* was first evaluated. Figure 1A shows the resulting growth curves in both shake flasks and the bioreactor. In the absence of a preculture, the growth curve exhibited an initial 4 h lag phase. This phase was minimized in both the STBR and shake-flask cultures inoculated from a preculture, where curves immediately entered the exponential phase, with an average OD_600_ value after 2 h of 1.36 and 1.02 for the bioreactor and the flask cultures, respectively. In comparison, the OD_600_ of the shake-flask culture without preculture after the same fermentation time was only 0.25. To evaluate culture performance under different cultivation conditions, biomass accumulation was monitored over time by sampling 50 mL aliquots from the bioreactor and the flask and was then normalized to the total volume (Figure 1B). Biomass in the STBR increased progressively, with the highest increase between 2 and 4 h, corresponding to the exponential growth phase. Interestingly, after 6 h of fermentation, the sample cultured in the flask did not show a similar biomass value (0.74 g/L) compared to the bioreactor (1.8 g/L), although they showed similar OD_600_ values of 3.16 and 3.12, respectively. This difference in biomass value was possibly due to the more homogenous conditions offered by the STBR, in particular regarding temperature, agitation and aeration control. At 24 h, on the other hand, no statistical difference was observed between the two fermentations. To evaluate the process reproducibility, the coefficient of variation (CV) was calculated as the percentage ratio between the standard deviation (SD) and the mean of the three biological replicates. Biomass showed a CV of 14.19% and 15.83% for the STBR culture at 6 and 24 h, respectively, indicating a low variability across independent fermentation batches. The pH of the bioreactor culture increased over time, rising from approximately 7.0 at 2 h to 8.6 after 24 h. A similar pH increase was found in the shake-flask culture, which went from 6.7 at 2 h to 8.3 after 24 h. This pH variation was driven by the metabolic activity of the bacteria during the fermentation and could influence EV production by *S. epidermidis*, since pH is a known regulator of bacteria activity and can influence the vesiculation in different ways depending on the bacteria strain [48,49,50].

### 2.2. Dimension and Concentration of EVs

**Figure 2:**
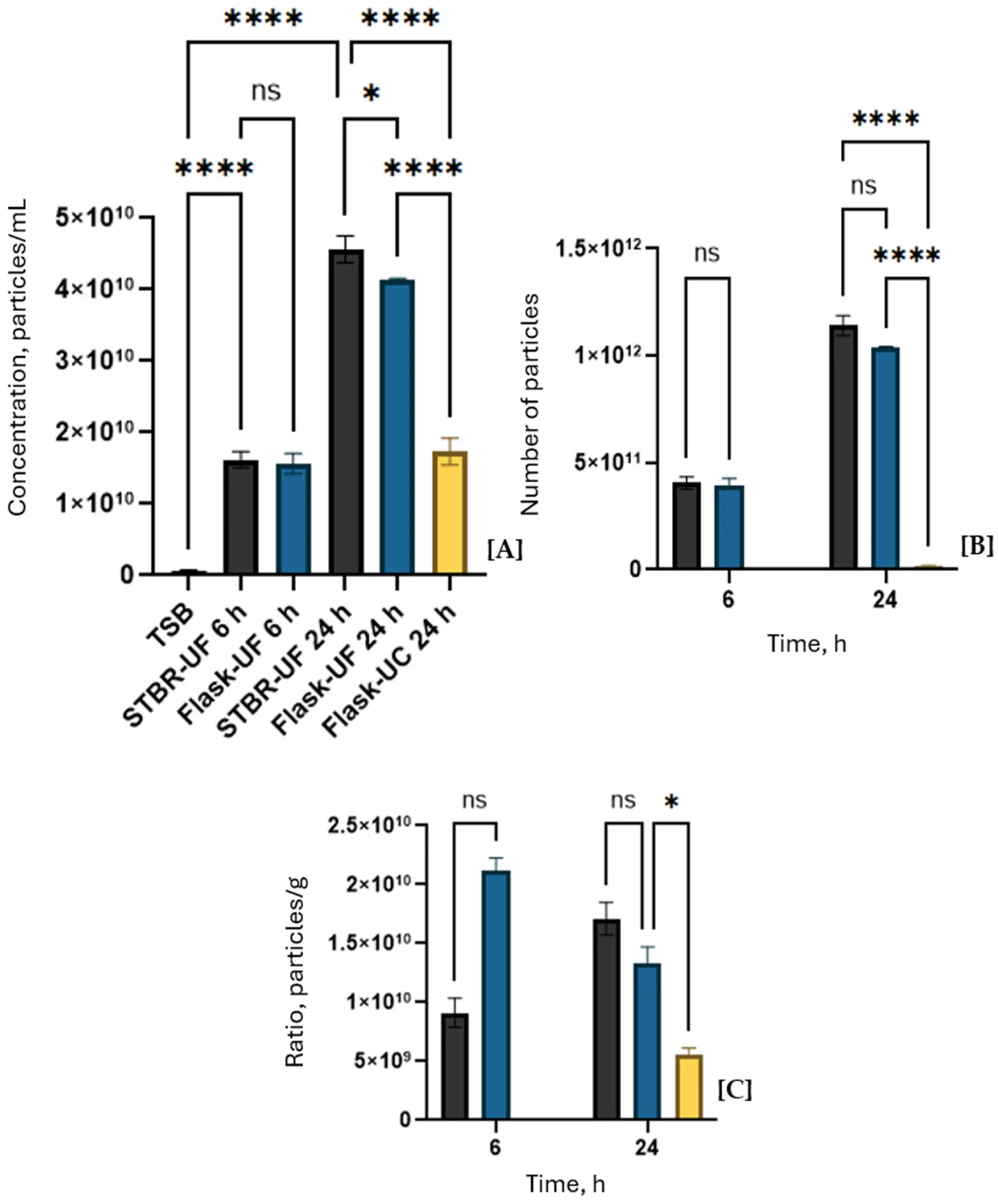
(**A**) Concentration of particles extracted via UC or UF, measured by NTA (ns, *p* > 0.05; *, *p* = 0.0194; ****, *p* < 0.0001). (**B**) Total particle yield recovered *via* UC or UF over time (ns, *p* > 0.05; ****, *p* < 0.0001), (**C**) concentration of particles normalized to biomass (ns, *p* > 0.05; *, *p* = 0.0287). 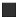 = STBR-UF; 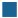 = Flask-UF; 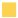 = Flask-UC.

To quantify EV production, the particle concentration was measured by nanoparticle tracking analysis (NTA) as shown in Figure 2A. The particle concentration increased over time for both culture systems. No statistical difference was observed between the flask and the STBR cultures purified via the dead-end UF method at 6 h (*p* = 0.9912), whereas a minor yet statistically significant difference was detected at 24 h (*p* = 0.0194). For the STBR-UF sample, the concentration increased from 1.62 × 10^10^ ± 1.13 × 10^9^ particles/mL at 6 h of fermentation (CV = 6.97%) to 4.56 × 10^10^ ± 1.87 × 10^9^ particles/mL after 24 h (CV = 4.1%). Similarly, the Flask-UF culture yielded 1.56 × 10^10^ ± 1.45 × 10^9^ particles/mL at 6 h (CV = 9.29%) and 4.14 × 10^10^ ± 1.21 × 10^8^ particles/mL at 24 h (CV = 0.29%). Interestingly, the concentration of particles isolated through UC (Flask-UC) was significantly lower than that of the samples purified via UF, reaching only 1.73 × 10^10^ ± 1.85 × 10^9^ particles/mL after 24 h of fermentation. For contrast, the Tryptic Soy Broth (TSB) control showed little to no particles in the NTA measurement, with an average value of 5.74 × 10^8^ and a particle/frame ratio of 8, which can be assumed as noise in the measurement. This concentration was approximately two orders of magnitude lower than that of STBR or flask samples, indicating that the contribution of medium-derived particles to the NTA signal was comparatively small. Figure 2B shows the total number of particles in the final processed volume. As can be seen, the UF strategy yielded a much higher time-dependent particle count, compared to the sample processed with UC, in both the fermentation setups. The particle concentration normalized to the biomass accumulation can be seen in Figure 2C. No statistical difference was found between the flask culture and the STBR, at 6 or 24 h. However, a minor yet statistically significant difference was found between the Flask-UC and the Flask-UF samples at 24 h of fermentation (*p* = 0.0287), confirming a higher recovery of the EV-enriched sample in the membrane-based isolation method.

**Figure 3:**
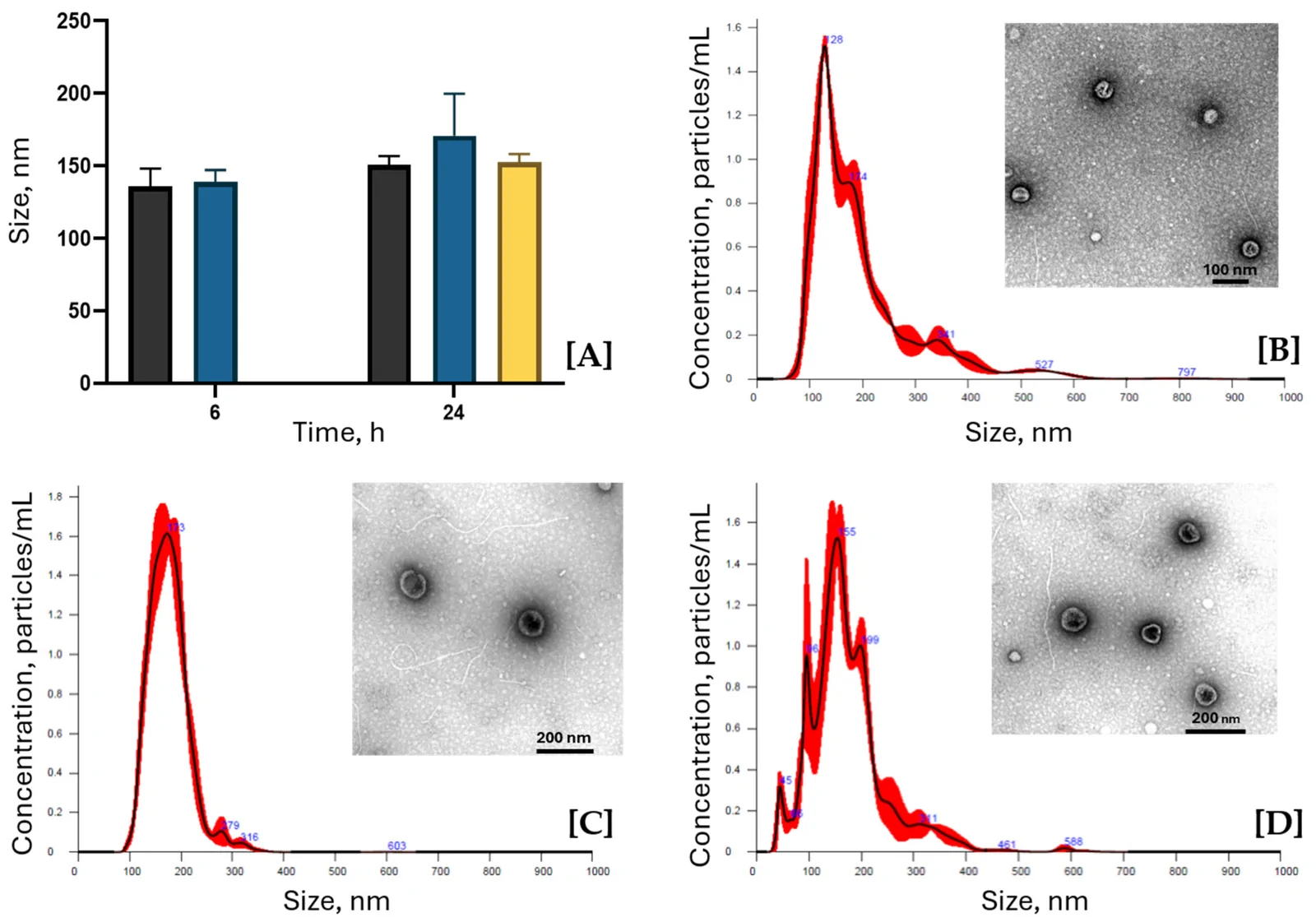
(**A**) Size distribution of particles isolated from different culture conditions with different extraction methods, after 6 and 24 h of fermentation (n = 3). TEM micrographs and NTA graphs of particles isolated from (**B**) STBR-UF 24 h, (**C**) Flask-UF 24 h and (**D**) Flask-UC 24 h. 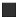 = STBR-UF; 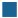 = Flask-UF; 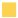 = Flask-UC.

To evaluate the impact of different conditions and isolation methods on the particle size and morphology, the size mode of the EV-enriched sample and the corresponding TEM micrographs were analyzed (Figure 3). For all time points, particles showed a consistent size distribution across all culture conditions (Figure 3A), with average modes of 135.6 ± 12.5 nm for STBR-UF and 138.8 ± 8.2 nm for Flask-UF after 6 h. A similar trend was observed across different extraction methods after 24 h, yielding sizes of 152.6 ± 5.5 nm for Flask-UC, 170.8 ± 28.9 nm for Flask-UF, and 150.4 ± 6.5 nm for STBR-UF. Furthermore, TEM micrographs confirmed the presence of vesicle-like structures in all samples, with comparable size and spherical morphology across all tested conditions.

### 2.3. Protein Quantification

**Figure 4:**
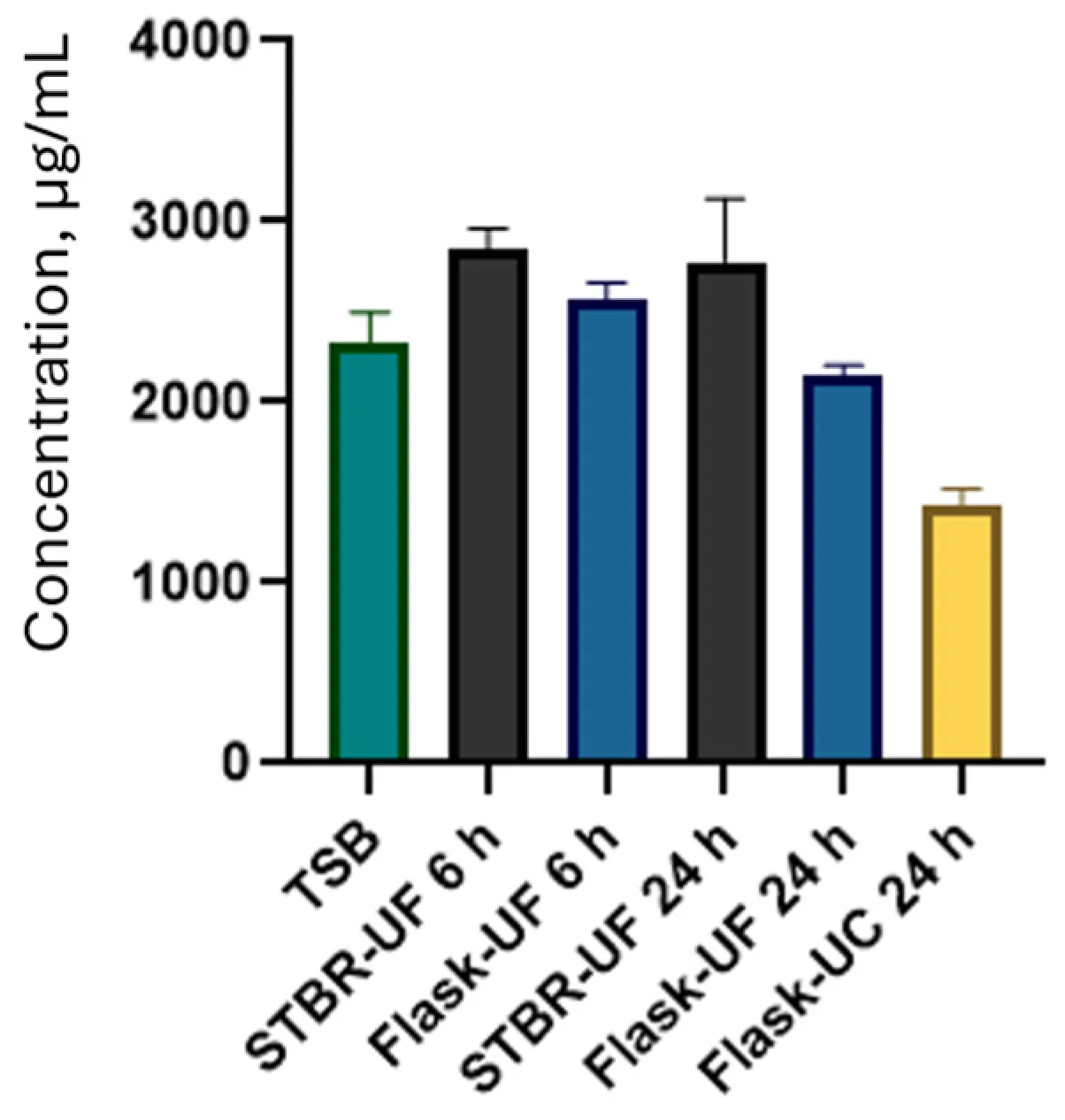
Averaged protein concentration of particles isolated from *S. epidermidis*. 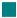 = TSB; 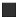 = STBR-UF; 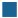 = Flask-UF; 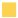 = Flask-UC.

The content of proteins in the EV-enriched sample was determined by BCA assay; see Figure 4. No significant differences in protein content were found between samples purified with UF at different time points or under the two different culture conditions (STBR-UF and Flask-UF). In contrast, Flask-UC yielded a significantly lower protein concentration (1436.67 ± 85.05 μg/mL) compared to all other samples. It is important to note that the TSB control resulted in a high protein content (2322.6 μg/mL), indicating a non-thorough depletion of soluble proteins in the processed EV-enriched sample.

### 2.4. EV Composition

**Figure 5:**
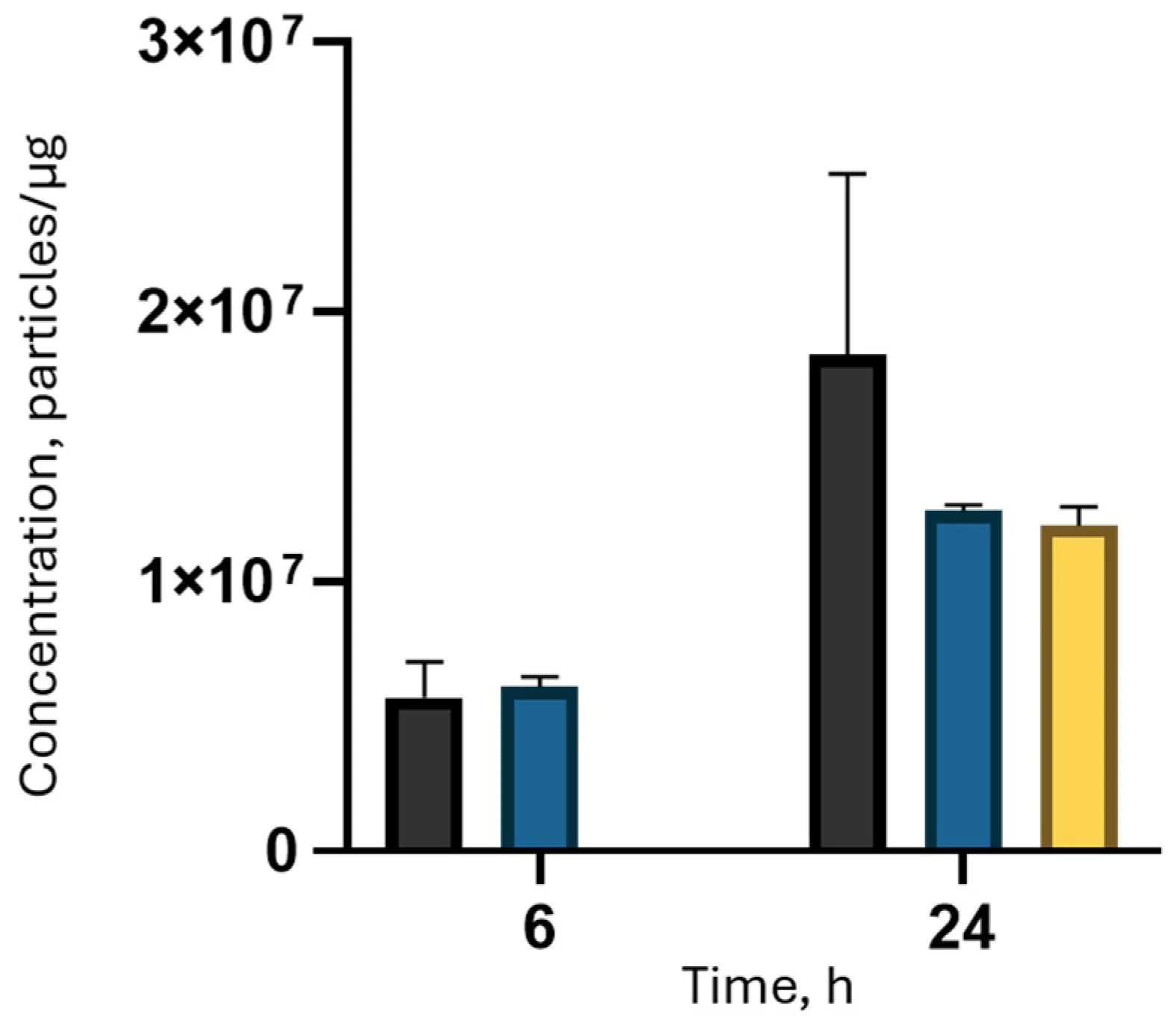
Particle to protein ratio of EV-enriched samples for particle enrichment evaluation. 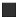 = STBR-UF; 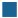 = Flask-UF; 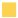 = Flask-UC.

As a preliminary indicator of sample composition, the particle-to-protein ratio was calculated by dividing the particle concentration measured by NTA by the total protein concentration determined by BCA assay. This metric provides an indirect estimate of the relative enrichment of particles over total protein content, although it should not be interpreted as a direct measure of the EV purity [51]. Figure 5 shows the particle-to-protein ratios calculated for the different conditions. At equivalent fermentation time, UF-derived isolates displayed particle-to-protein ratios comparable to those obtained via UC. This result suggests that the UF-based purification did not markedly increase the relative protein burden in the final preparations, thereby enabling an efficient recovery process.

## 3. Discussion

The production and characterization of EVs from Gram-positive bacteria remain less extensively investigated than those from Gram-negative ones, largely due to differences in cell membrane and cell wall architecture and the lack of standardized and scalable isolation protocols. In this study, we developed and evaluated a production strategy compatible with a process scale-up for the cultivation of *S. epidermidis* in a stirred-tank bioreactor and compared two downstream recovery methods, i.e., ultracentrifugation and ultrafiltration, in terms of particle yield, size and morphology, protein content and particle enrichment. Our findings show that the STBR fermentation combined with a preculture resulted in a minimization of the growth lag-phase. This lag phase can be attributed to the physiological adaptation required following transfer from a solid culture environment to growth in liquid medium [52]. A preculture, on the other hand, provides cells that are already metabolic active and adapted to the growth medium. In any case, once the adaptation period ends, the cells rapidly enter the exponential phase and are able to compensate for the initial delay, reaching comparable OD_600_ values to the precultured fermentation. Furthermore, the bioreactor enhanced biomass accumulation compared with standard shake-flask cultures during the exponential phase, even though no significant difference was found in the cultures measured in the stationary phase. Mixing efficiency and dissolved oxygen are known to influence bacterial physiology and vesiculation [53,54], and an improved control over these parameters could have had an effect over biomass accumulation, especially during the exponential phase where bacteria are the most metabolically active. On the other hand, during the stationary phase cell growth is primarily limited by nutrient depletion and the accumulation of metabolic by-products, and the fermentation reaches an equilibrium [55]. Unlike shake flasks, where oxygen supply and pH could not be continuously monitored or controlled, the STBR provided a more controlled environment, resulting in more consistent growth rate and reduced batch-to-batch variability [56]. These findings are consistent with previous studies showing that controlled fermentation systems offer clear advantages for processes involving Gram-negative bacteria [22,57], whereas comparable evidence remains limited for Gram-positive ones. In this work, we also performed a side-by-side comparison of UC and UF for EV isolation and purification to assess the scale-up feasibility of the two strategies. Consistent with previous works [28,58,59], UF yielded a significantly higher particle concentration than UC. Importantly, this difference was maintained after accounting for the different final sample volumes, with UF resulting in a higher total number of recovered particles than UC. Furthermore, a time-dependent increase in particle concentration was observed between 6 and 24 h. The following two timepoints were selected to represent two distinct physiological stages of the fermentation: the late exponential and the stationary phase, respectively. This selection was also supported by previous studies reporting a phase-dependent difference in bacterial EV production [60]. This result is consistent with the known limitations of UC, as high *g*-forces during UC can cause vesicle aggregation, membrane disruption, or inefficient pelleting of smaller particles, leading to yield loss [31,61]. In contrast, UF operates under milder conditions and is less likely to promote vesicle aggregation, thereby enabling a more efficient recovery [26,58]. However, vesicles may adsorb onto the membrane filter in a dead-end configuration, which may cause progressive fouling and limit recovery over time. To mitigate this phenomenon, optimized membrane rinsing, diafiltration, or transition to a tangential-flow configuration may be required [62]. Notably, our NTA results show that particles isolated by both methods displayed comparable size distributions, consistent with the size range reported for *S. epidermidis* EVs in previous studies (146–172 nm) [47,63]. However, NTA does not distinguish between EVs and other particles of comparable size, thus more in-depth analysis of the nature of these particles could be performed in the future. For instance, TEM micrographs confirmed the presence of vesicle-like particles with a visible lipid bilayer and intact spherical morphology, indicating that neither procedure induced major structural changes. Nevertheless, the slightly larger size observed in UF-isolated samples may reflect the co-isolation of protein aggregates, membrane fragments or other cell debris, which may affect the sample composition. In addition, evaluation of extracellular DNA, RNA or peptidoglycan may be performed in future work to evaluate the presence of contaminants derived from cell lysis. Protein contamination is a common issue in many different isolation methods, including UC [64], and introducing a further purification step, such as size-exclusion chromatography (SEC), or implementing a TFF with diafiltration could help preserve EV recovery and improve particle enrichment [27,65]. Furthermore, a TFF configuration would enable us to process larger volumes compared to the dead-end system implemented in this work, due to the intrinsic limitation of the latter. Future studies will also need to assess particle stability over time and throughout the cultivation process. The increase in culture pH observed in the STBR over the 24 h fermentation may also be relevant for EV concentration and size distribution, as pH has been reported to modulate EV biogenesis in several bacterial species. Future research may be necessary to examine whether maintaining the pH close to neutrality affects EV yield, protein content or cargo composition in *S. epidermidis*. Regarding the protein content of the samples, we found that EVs extracted via UF had a significantly higher amount of protein compared to those extracted from UC. This finding is consistent with the higher particle concentrations measured by NTA, suggesting an improved recovery of particles in the EV-enriched preparation through the UF process. Furthermore, this result may reflect protein loss during UC, potentially associated with vesicle disruption, aggregation or repeated washing steps. Nonetheless, the higher value of the UF samples may also indicate the retention of soluble proteins, cell debris or protein aggregates still present in the samples. Protein concentration alone cannot be considered an indicator of EV quality or recovery but should always be interpreted together with other measurements, like particle concentration, total particle yield and other complementary characterization techniques. Therefore, a more thorough purification protocol may be employed to further reduce soluble protein carryover and better control the composition of the recovered EV-enriched samples. Furthermore, in-depth evaluation of the presence of extracellular DNA, RNA or peptidoglycan could be performed in future works to better assess the nature of the recovered particles and exclude the possible contamination from lysed cells. In any case, particle-to-protein ratio, used here as a preliminary indicator of particle enrichment rather than a direct measure of purity, was comparable between UC- and the UF-derived samples after 24 h of fermentation. This result suggests that both methods can recover EV-enriched samples, making UF a feasible alternative to UC under the conditions tested. It is important to point out that the present study represents an initial step toward the development of a scalable bacterial EV production strategy, rather than a validation of a fully scaled process. The transition from a 200 mL shake-flask culture to a 2 L controlled STBR-enabled evaluation of EV production under controlled culture conditions at increased working volume. Furthermore, this transition also involved a change in the fermentation environment and mixing specifics. In the STBR mixing is driven by mechanical impellers submerged in the culture media, and not just by orbital agitation, allowing for an overall more homogenous fermentation. Similarly, the stirred-cell UF experiments demonstrated the feasibility of membrane-based EV concentration as an alternative to UC. Nevertheless, the 50 mL dead-end configuration used here is inherently limited for processing larger volumes. Future scale-up should therefore investigate TFF systems with increased membrane area and controlled transmembrane pressure, together with quantitative assessment of EV recovery, membrane fouling, processing time, and product quality across scales.

Overall, this study provides one of the first evaluations of integrated upstream and downstream process conditions for the reproducible and scalable production of Gram-positive bacterial EVs, and, to our knowledge, the first focused on *S. epidermidis*. By validating that controlled STBR fermentation can be successfully combined with UF-based recovery, we established a more time-efficient procedure that is better suited for processing higher volumes than traditional UC-based methods and shake-flask cultures. These findings address the increasing demand for scalable and reproducible manufacturing procedures for bacterial EVs and support their further development as platforms for emerging therapeutic and biotechnological applications.

## 4. Materials and Methods

### 4.1. Microorganism and Growth Conditions

*S. epidermidis* ATCC 12228 was cultured overnight on Tryptic Soy Agar (TSA, ThermoScientific™ Oxoid™, Basingstoke, Hants, UK) at 37 °C, and it was used to prepare a preculture as follows. The culture was resuspended in sterile Tryptic Soy Broth (TSB, ThermoScientific™ Oxoid™, Basingstoke, Hants, UK) and the Optical Density at 600 nm (OD_600_) was checked with a Lambda 465 spectrophotometer (PerkinElmer^®^, Waltham, MA, USA). The suspension was diluted with TSB until the value of absorbance reached between 0.8 and 1. Then, 20 mL of the diluted suspension was added to 180 mL of sterile TSB in a 500 mL Erlenmeyer flask (to set the inoculum at 10% *v*/*v*) closed with a cotton cap. The culture was finally incubated at 37 °C under a constant agitation of 125 rpm. Once it reached the exponential growth phase (4–6 h), with an OD_600_ of around 1.5, it was used to inoculate a flask and an STBR (see Section 4.2). As comparison, one flask was prepared with the same procedure as above but with a fermentation time of 24 h to investigate the effect of the preculture on bacterial growth rate.

### 4.2. Cultures in Flask and Bioreactor

The preculture was diluted in TSB until the OD_600_ reached between 0.8 and 1. For the shake flask culture, 20 mL of this suspension was added to 180 mL of culture media in Erlenmeyer flask (to set the inoculum at 10% *v*/*v*), closed with a cotton cap. The culture was incubated at 37 °C, under a constant agitation of 125 rpm. For the scale up of the EV production, the fermentation was performed in the RALF STBR (Bioengineering^®^, Wald, Switzerland), characterized by a total volume of 3 L and equipped with an external jacket for the temperature monitoring and a shaft with two six-blades-radial “Rushton” turbines. A total of 200 mL of the preculture was added to 1800 mL of sterile TSB in the bioreactor and then kept at 37 °C via the external jacket and under a constant 125 rpm stirring. The pO_2_ was set to 60% and a sparger at the bottom of the reactor intermittently fluxed air into the culture to keep the pO_2_ level at 60%. The pH of the STBR culture was monitored through the pH-meter of the reactor, while for the shake-flask culture an aliquot was taken at each time point and the pH was measured through an external pH-meter. Three independent fermentations (n = 3) were performed for each different EV sample and used for further characterizations.

### 4.3. Growth Curve and Biomass Evaluation

The growth curves of *S. epidermidis* in flask and bioreactor were determined by taking a 2 mL aliquot of the cultures at different timepoints and measuring the OD_600_. TSB was used as blank. The biomass accumulation of the culture inside the bioreactor and the flask was evaluated over time by measuring the dry weight, as follows. A 50 mL aliquot was taken at different timepoints and centrifuged (Centrifuge 5810R, Eppendorf^®^, Hamburg, Germany) at 4500 rpm for 25 min at 4 °C. The pellet was washed once by resuspending it in PBS and then centrifuging it at the same conditions as before. Subsequently, the pellet was dried on a thermobalance (MB 120, Ohaus^®^, Parsippany, NJ, USA) to obtain dry weight. The supernatant was kept in the fridge at 4 °C and used for EV isolation or stored at −80 °C. For the flask samples, one flask was prepared for each time point and used specifically for the respective biomass evaluation.

### 4.4. EV Extraction and Purification

EV extraction method was based on Zaborowska et al., with slight modifications [63]. For both the STBR and flask cultures, the supernatant obtained through the centrifugation at the 6 h and 24 h time point was first microfiltered with a 0.45 µm cellulose acetate membrane filter and the permeate was then microfiltered with a 0.22 µm filtration system (Steriflip^®^ Millipore^®^, Burlington, MA, USA) to remove all the remaining cell debris. In order to concentrate and purify the EVs, 50 mL of the suspension was ultrafiltered in a 50 mL Amicon^®^ stirred cell with a 44.5 mm diameter membrane filter with a MWCO of 100 kDa (Millipore^®^), under a constant stirring of 100 rpm and a pressure of 2 bar, until half of the volume was filtered (Flask-UF). As control, 50 mL of sterile TSB were processed as described above and stored at 4 °C until further characterizations. For the UC comparison, after the two microfiltrations, 50 mL of the supernatant deriving from the same flask culture was filtered in a Vivaspin^®^ concentrator with a 100 kDa cutoff at 6000× *g* for 15 min, and then the retentate was ultracentrifuged at 150,000× *g* for 1 h at 4 °C. The pellet was washed in PBS once and ultracentrifuged with the same conditions described above. The final EV sample (here called Flask-UC) was resuspended in 1 mL of PBS and stored at −80 °C until use.

### 4.5. Characterizations

#### 4.5.1. NTA

The concentration and size distribution of EVs were examined by NTA using NanoSight NS300 (Malvern Instruments, Worcestershire, UK). For each independent EV replicate, samples were diluted in PBS as needed (typically 1:100 or 1:200) to achieve a final concentration of 30–80 particles per frame. Measures of a duration of 60 s were performed in triplicate at 25 °C at a flow rate of 50 (arbitrary units) with the syringe pump. The Camera Level was set between 14 and 16. Analysis settings were adjusted to a Detection Threshold between 4 and 8. Data outputs were generated using NanoSight NTA Software v3.4.

#### 4.5.2. Protein Quantification

An aliquot of each EV sample was kept for protein evaluation using the Novagen^®^ BCA kit (Merck, Milan, Italy), following the manufacturer’s instructions. Each independent sample was measured in triplicate and BSA dilutions were used as standard to build the calibration curve (R^2^ = 0.9964).

#### 4.5.3. TEM

The morphology of EVs was investigated with transmission electron microscopy (TEM), using a JEM 1400 Flash (JEOL, Tokyo, Japan) operating at 80 kV, as described before [66]. Briefly, a drop of 7 μL of the sample was deposited on carbon and Formvar-coated 400 mesh copper grids (Ted Pella, Redding, CA, USA), previously glow-discharged with an Edwards E12E vacuum coating unit (Edwards Vacuum, Burgess Hill, UK). After 5 min of incubation to allow the adsorption of EVs, the grids were rinsed several times with water and negatively stained with aqueous 0.5% *w*/*v* uranyl acetate. The excess solution was then removed with filter paper.

#### 4.5.4. Statistical Analysis

Statistical analyses were carried out using Graphpad Prism, version 11.0.2 (GraphPad, San Diego, CA, USA). Significant differences were determined by *t*-test, One-way Analysis of Variance (ANOVA), Two-way ANOVA, or Tukey’s multiple comparison test. All data were expressed as means ± standard deviations (SDs), when applicable. Differences were considered statistically significant at *p* < 0.05. Batch-to-batch variability was assessed by calculating the coefficient of variation (CV) across independent biological replicates, as the ratio between the standard deviation and mean, expressed as a percentage.

## 5. Conclusions

In conclusion, this study demonstrates the feasibility of combining controlled stirred-tank bioreactor cultivation with ultrafiltration for the production and recovery of *S. epidermidis* EVs. The STBR supported bacteria cultivation at a larger working volume and resulted in higher biomass during the early growth phase, while UF provided higher particle recovery than UC under the conditions investigated. These findings support the proposed workflow as a promising first step toward more reproducible and scalable EV production for Gram positive bacteria. Nonetheless, the present study does not establish full process scalability, and it does not assess EV-specific purity, as engineering scale-up parameters and comprehensive molecular characterization were not evaluated. Further studies should therefore evaluate scale-dependent parameters and focus on TFF-based downstream processing, together with more thorough characterization of EV identity, purity, stability, and biological activity.

## Data Availability

Data are contained within the article.

## Supplementary Materials

The following supporting information can be downloaded at https://www.mdpi.com/article/10.3390/ijms27177797/s1.
