# Supplementary material for: Towards a Scalable Production of Staphylococcus epidermidis Extracellular Vesicles: Integration of Stirred-Tank Bioreactor and Ultrafiltration-Based Recovery
Source: Int J Mol Sci. 2026 Aug 31;27(17):7797. doi: 10.3390/ijms27177797 (PMC13566652; doi:10.3390/ijms27177797)
Supplement: Supplementary file 1 [file ijms-27-07797-s001.zip › ijms-4452618-supplementary.pdf]

Supporting Information

Hydrodynamic stress estimates for ultrafiltration and ultracentrifugal sedimentation of a 150 nm vesicle

We here report the assumptions, equations, and numerical checks used to compare published shear-stress values for ultrafiltration or tangential-flow filtration with hydrodynamic stresses associated with ultracentrifugal sedimentation. The quantities are not strictly equivalent: filtration studies generally report a process-level or membrane-wall shear stress, whereas the ultracentrifugation calculation below resolves the idealized traction on a sedimenting particle.

Kaya et al. [34] studied membrane-surface shear stresses of 1.33 and 1.48 Pa in a vibratory hollow-fibre membrane bioreactor. These values are system-specific and are included only as examples of low-pascal membrane-surface stresses; they are neither a universal ultrafiltration range nor extracellular-vesicle-specific values.

In an extracellular-vesicle-specific tangential-flow filtration study, Luo et al. [35] reported that excessive shear stress above 9.05 Pa led to protein aggregation and damage to small extracellular vesicles. However, this value is used below only as a published comparison level, not as a universal damage threshold. The input parameters and the main assumptions made in the calculation are shown in Table S1.

Table S1. Assumptions and input values.

Parameter	Assumption or value
Particle geometry	Rigid, no-slip sphere; diameter D_p = 150 nm; radius R_p = 75 nm.
Particle density	ρ_P= 1045 kg m^{-3}, used as a representative value for empty liposomes measured by analytical ultracentrifugation [36]. This value is formulation-specific and is not universal for liposomes or extracellular vesicles.
Continuous phase	Water at 25 °C: ρ_F= = 997.047 kg m^{-3} and dynamic viscosity μ_F = 0.890 × 10^{-3} Pa s.
Centrifugal field	Local acceleration of 100,000 × g (a = 9.80665 × 10^5 m s^{-2}).
Dilute calculation	Newtonian fluid; terminal settling; no wall effects; no hydrodynamic or mechanical interactions with other particles; no particle deformation.
Concentrated calculation	The Richardson–Zaki relation with n = 4.65 is used as an illustrative low-Reynolds-number hindered-settling correlation [37]. It is not validated here for Brownian, deformable, or polydisperse vesicles.
Other assumptions	The model does not include adhesion, contact mechanics, pellet permeability, fluid drainage through a pellet, or stresses generated during pellet resuspension.

2. Dilute-system calculation

In this section, the hydrodynamic stresses associated with ultracentrifugal sedimentation are evaluated for a dilute solid–liquid suspension, in which particle–particle interactions can be neglected.

The difference in mass density between the solid dispersed phase and the liquid continuous phase is:

$$\Delta\rho = \rho_P - \rho_F = 47.953 \text{ kg m}^{-3}$$

while the particle volume is:

$$V = \frac{4}{3}\pi R^3 = 1.767\times10^{-21} \text{ m}^3$$

It follows that the buoyancy-corrected centrifugal force can be written as,

$$F_c = \frac{4}{3}\pi R^3 \Delta\rho\, a = 8.310\times10^{-14} \text{ N}$$

Accordingly, the terminal velocity derived from Stokes' drag law is,

$$v_t = \frac{F_C}{6\pi\mu_F R_P} = \frac{2R_P^2 \Delta\rho\, a}{9\mu_F} = 6.605\times10^{-5} \text{ m s}^{-1}$$

At terminal motion, the drag force is: $F_D = 6\pi\mu_F R_P v_t = 8.310\times10^{-14}$ N. The particle Reynolds number is small (Re≅10^{-5}) and supports use of the creeping-flow approximation. Assuming that the continuous phase behaves as a Newtonian fluid, the tangential surface shear stress for a rigid no-slip sphere is:

$$\sigma_{r\theta} = \frac{3\mu_F v_t}{2R_P}\sin\theta$$

The maximum magnitude occurs at the equator; thus, $\sigma_{r\theta,max} = \frac{3\mu_F v_t}{2R_P} = 1.176$ Pa. The surface-area-averaged magnitude of the tangential shear stress is:

$$\langle\sigma_{r\theta}\rangle = \frac{1}{4}\pi R_P^2 \int_S |\sigma_{r\theta}|\, dS = \frac{\pi}{4}\sigma_{r\theta,max} = 0.923 \text{ Pa.}$$

The drag-equivalent stress is defined as:

$$\sigma_D = \frac{F_D}{4\pi R_P^2} = 1.176 \text{ Pa}$$

For the isolated Stokes sphere, σ_D is numerically equal to $\sigma_{r\theta,max}$. It is not the area-averaged tangential shear stress, because the total drag contains both pressure and viscous-traction contributions.

3. Concentrated suspension: free sedimentation and fixed-speed benchmark

In this section, the hydrodynamic stresses associated with ultracentrifugal sedimentation are evaluated for a concentrated solid–liquid suspension. For this purpose, we use the Richardson–Zaki relation that describes the dependence of the hindered-settling velocity on the particle volume fraction (ϕ) by relating the settling velocity in a concentrated suspension (v_ϕ) to the terminal velocity of an isolated particle (v_t),

$$\frac{v_\phi}{v_t} = (1-\phi)^n$$

where n= 4.65. An effective scalar resistance factor can be inferred from this relation:

$$K(\phi) = \frac{v_t}{v_\phi} = (1-\phi)^{-4.65}$$

For freely settling particles at terminal velocity, provided no load is carried by particle contacts, the scalar drag balance is,

$$F_{D,\phi} = 6\pi\mu_F R_P v_\phi K(\phi) = 6\pi\mu_F R_P v_t = F_C$$

Thus, within this hindered-settling representation, concentration lowers the settling velocity but does not increase the net terminal drag per particle as shown in Table S2. Also, it does not provide the local distribution of tangential stress on a particle surface.

Table S2. Hindered-settling calculation and formal fixed-speed comparison.

**Particle volume fraction, ϕ**	$K(\phi)$	**Free-settling velocity v_ϕ, micrometres s^{-1}**	**Free-settling drag-equivalent stress σ_D, Pa**	**Fixed-speed drag-equivalent stress, $\sigma_{D,fixed}$, Pa**
0	1.000	66.048	1.176	1.176
0.050	1.269	52.032	1.176	1.492
0.100	1.632	40.465	1.176	1.919
0.200	2.823	23.401	1.176	3.318
0.300	5.252	12.577	1.176	6.174
0.355	7.698	8.580	1.176	9.050

4. Sedimentation and pellet formation

If the dilute particle-fluid slip velocity v_t were artificially held constant while the scalar resistance factor $K(\phi)$ increased, the formal drag-equivalent stress would be $\sigma_{D,fixed} = \sigma_D K(\phi)$. Solving $\sigma_{D,fixed}$ = 9.05 Pa gives,

$$\phi_{cr} = 1 - \left(\frac{\sigma_D}{9.05}\right)^{1/4.65} = 0.355 \text{ (35.5\% v/v)}$$

Using the surface-area-averaged tangential shear magnitude, rather than the drag-equivalent metric, in the same formal scaling would give ϕ_{cr} = 0.388. This dependence on the selected stress metric further shows that neither value is a physical concentration threshold for freely sedimenting vesicles.

Accordingly, the value 0.355 is retained only as the result of a fixed-speed drag thought experiment. During free hindered sedimentation, v_ϕ decreases as $K(\phi)$ increases and the net terminal drag remains equal to F_c.

Once a load-bearing pellet or a strongly compacted layer forms, the free-settling force balance no longer describes the complete mechanical state. Near-field lubrication forces become important as liquid is displaced from narrow interparticle gaps, and direct contact, adhesion, vesicle deformation, and, if present, frictional interactions can transmit additional normal and tangential loads [38,39]. These mechanisms are not represented by the Richardson–Zaki scalar correction.

Additional local stresses could therefore exceed 9.05 Pa, including at nominal or bulk particle volume fractions below 0.355. This possibility cannot be quantified from particle volume fraction alone. A predictive calculation would require at least the local interparticle gaps and relative velocities, spatial packing distribution, pellet thickness and permeability, vesicle elastic properties, and an appropriate contact, adhesion, or friction law. High-speed centrifugation has independently been shown to induce extracellular vesicle aggregation [31].

5. Summary of verified values and interpretation

The quantitative comparison between UF and UC is summarized in Table S3. Under the assumptions adopted for the UC calculation, a 150 nm vesicle modelled as a rigid no-slip sphere sedimenting in water at 25 °C and 100,000×g experiences a maximum tangential surface shear stress of approximately 1.18 Pa and an area-averaged tangential shear stress of 0.92 Pa. These values are comparable to membrane-surface shear stresses of 1.33-1.48 Pa reported under relatively mild hollow-fibre filtration conditions and are lower than the reported TFF comparison level of 9.05 Pa, above which protein aggregation and sEV damage were observed. In a concentrated suspension undergoing free hindered sedimentation, particle crowding reduces the sedimentation velocity and thus does not, by itself, increase the terminal drag acting on each vesicle. However, the dilute Stokes-flow calculation does not account for short-range lubrication interactions, collisions, direct contacts, interparticle friction, vesicle deformation, or pellet compaction. All these phenomena may generate substantially higher local stresses during the later stages of ultracentrifugation; such stresses are not captured by the calculations presented above.

Table S3. Consistency-checked results.

Condition or quantity	Verified value	Interpretation
Vibratory hollow-fibre membrane filtration	1.33-1.48 Pa	Published, system-specific membrane-surface values; not EV-specific [34]
EV-specific TFF	>9.05 Pa	Reported detrimental regime for protein aggregation and sEV damage [35]
UC, dilute terminal velocity	66.048 μm s^{-1}	Rigid 150 nm sphere in water at 25 °C and 100,000 × g
UC, dilute drag force	8.310 × 10^{-14} N	Equals the buoyancy-corrected centrifugal force at terminal motion
UC, maximum tangential surface shear	1.176 Pa	Exact isolated-sphere Stokes-flow result
UC, area-average magnitude of tangential shear	0.923 Pa	Surface average of $\lvert\sigma_{r\theta}\rvert$; distinct from $\frac{F_D}{4\pi R_p^2}$
UC, drag-equivalent stress	1.176 Pa	Scalar metric $\frac{F_D}{4\pi R_p^2}$; numerically equals $\sigma_{r\theta,max}$ for an isolated Stokes sphere
UC, freely sedimenting concentrated suspension	Net terminal drag unchanged in the scalar R-Z balance	Settling velocity decreases with ϕ; local surface shear is not determined by this correlation
UC, pellet or compaction stage	Not determined	Additional local stresses may be larger but cannot be calculated from ϕ alone

References

[31] Linares, R.; Tan, S.; Gounou, C.; Arraud, N.; Brisson, A. R. High-Speed Centrifugation Induces Aggregation of Extracellular Vesicles. Journal of Extracellular Vesicles 2015, 4, 29509. doi: 10.3402/jev.v4.29509.

[34] Kaya, R.; Ersahin, M. E.; Ozgun, H.; Tarabara, V.; Koyuncu, I. Membrane Fouling in Vibratory MBRs: Impact Distance Complements Wall Shear Stress as a Fouling Predictor. Water Environment Research 2026, 98(4), e70362. doi: 10.1002/wer.70362.

[35] Luo, L.; Du, Y.; Niu, X.; Liu, J.; Yuan, J.; Xu, H.; Wang, Y.; Li, H.; Li, Q. Optimized Tangential Flow Filtration for Efficient Isolation of Highly Pure and Bioactive sEVs. Analytical Chemistry 2025, 97(31), 17261-17274. doi: 10.1021/acs.analchem.5c03781.

[36] Mehn, D.; Iavicoli, P.; Cabaleiro, N.; Borgos, S. E.; Caputo, F.; Geiss, O.; Calzolai, L.; Rossi, F.; Gilliland, D. Analytical Ultracentrifugation for Analysis of Doxorubicin-Loaded Liposomes. International Journal of Pharmaceutics 2017, 523(1), 320-326. doi: 10.1016/j.ijpharm.2017.03.046.

[37] Richardson, J. F.; Zaki, W. N. Sedimentation and Fluidisation: Part I. Transactions of the Institution of Chemical Engineers 1954, 32, 35-53. Reprinted in Chemical Engineering Research and Design 1997, 75, S82-S100. doi: 10.1016/S0263-8762(97)80006-8.

[38] Yeo, K.; Maxey, M. R. Simulation of Concentrated Suspensions Using the Force-Coupling Method. Journal of Computational Physics 2010, 229(6), 2401-2421. doi: 10.1016/j.jcp.2009.11.041.

[39] Seto, R.; Mari, R.; Morris, J. F.; Denn, M. M. Discontinuous Shear Thickening of Frictional Hard-Sphere Suspensions. Physical Review Letters 2013, 111, 218301. doi: 10.1103/PhysRevLett.111.218301.
